# Cyanobacteria-Mediated Immune Responses in Pepper Plants against *Fusarium* Wilt

**DOI:** 10.3390/plants11152049

**Published:** 2022-08-05

**Authors:** Amer Morsy Abdelaziz, Mohamed S. Attia, Marwa S. Salem, Dina A. Refaay, Wardah A. Alhoqail, Hoda H. Senousy

**Affiliations:** 1 Botany & Microbiology Department, Faculty of Science, Al-Azhar University, Cairo 11884, Egypt; 2 Botany & Microbiology Department, Faculty of Science, Al-Azhar University (Girls Branch), Cairo 11884, Egypt; 3 Botany Department, Faculty of Science, Mansoura University, Mansoura 35516, Egypt; 4 Department of Biology, College of Education, Majmaah University, Majmaah 11952, Saudi Arabia; 5 Botany and Microbiology Department, Faculty of Science, Cairo University, Giza 12613, Egypt

**Keywords:** pepper, *Fusarium*, cyanobacteria, antioxidant enzymes, plants immunity, salicylic acid

## Abstract

Research in plant pathology has increasingly focused on developing environmentally friendly, effective strategies for controlling plant diseases. Cyanobacteria, including *Desmonostoc muscorum*, *Anabaena oryzae*, and *Arthrospira* *platensis*, were applied to *Capsicum annuum* L. to induce immunity against *Fusarium* wilt. Soil irrigation and foliar shoots (FS) application were used in this investigation. The disease symptoms, disease index, osmotic contents, total phenol, Malondialdehyde (MDA), hydrogen peroxide (H_2_O_2_), antioxidant enzymes (activity and isozymes), endogenous hormone content, and response to stimulation of defense resistance in infected plants were assessed. Results demonstrated that using all cyanobacterial aqueous extracts significantly reduced the risk of infection with *Fusarium oxysporum*. One of the most effective ways to combat the disease was through foliar spraying with *Arthrospira platensis*, *Desmonostoc muscorum*, and *Anabaena oryzae* (which provided 95, 90, and 69% protection percent, respectively). All metabolic resistance indices increased significantly following the application of the cyanobacterial aqueous extracts. Growth, metabolic characteristics, and phenols increased due to the application of cyanobacteria. Polyphenol oxidase (PPO) and peroxidase (POD) expressions improved in response to cyanobacteria application. Furthermore, treatment by cyanobacteria enhanced salicylic acid (SA) and Indole-3-Acetic Acid (IAA) in the infected plants while decreasing Abscisic acid (ABA). The infected pepper plant recovered from *Fusarium* wilt because cyanobacterial extract contained many biologically active compounds. The application of cyanobacteria through foliar spraying seems to be an effective approach to relieve the toxic influences of *F. oxysporum* on infected pepper plants as green and alternative therapeutic nutrients of chemical fungicides.

## 1. Introduction

The food issues worldwide result from the increased human population, besides plant pathogens resulting in whole or partial harm to crop yields [1]. Most roots of the *Solanaceae* family and other plants have suffered from soil fungal plant pathogens that cause harmful effects on morphological, physiological, molecular, and yield properties. Pepper (*Capsicum annuum* L.) is a hardy plant cultivated extensively worldwide. The annual production of Egyptian pepper is 623,221 tons, with a total cultivation area of 41,047 hectares [2]. Pepper crops worldwide, including in Egypt, are being destroyed by soil-borne diseases such as *F. oxysporum*, which causes significant losses in quantity and quality [3]. Despite the effectiveness of synthetic fungicides in eliminating *Fusarium* and minimizing the harmful effects, the ecological troubles and the increased fungal resistance to these chemical fungicides are evident. We should not fail to note that the excessive use of pesticides has led to more serious problems than the disease itself, as it has negatively affected humans, animals, the environment, and healthy microbial communities in soil and plants [4,5,6]. Therefore, biological control of *Fusarium* wilt through different non-pathogenic microorganisms such as cyanobacteria, fungi, yeast, and bacteria are good techniques [7,8]. Plant resistance means preventing or limiting the progression of its damage, whether biotic or abiotic [9,10]. Biological agents can induce systemic pepper plant resistance. Inducers of resistance affect anatomical structures, morphology, or the making of certain chemical composites that obstruct the pathogen or minimize the severity of stress [11,12]. Structure and chemical weapons may be present in the plant regardless of whether a pathogen is attacking it. These weapons may also originate from an attack on the plant by a pathogen or stress [13]. The destruction of *F. oxysporum* in-vitro and in vivo is similar to the activity of mancozeb chemical fungicides through inhibition of fungal growth and sporulation [14].

Algae, including cyanobacteria, act as bio-protectants and bio-stimulants for crop enhancement [15,16] by destructing the structure or function of the membrane of plant pathogens, devastating pathogenic enzymes, and the defeat of protein synthesis [17]. *Desmonostoc muscorum* is an effective bio-fungicide to control some plant pathogens such as *Alternaria porri* in-vitro [18]. It also can inhibit the radial *Fusarium* mycelial growth [19]. On the other hand, *Arthrospira platensis* has antifungal activity against *F. oxysporum* through polyphenols production in in-vitro [20] and in-vivo against *Moringa Fusarium* wilt [21]. *Arthrospira platensis* extract contains phenolics resulting in antifungal activity [22]. *Anabaena*, when applied to seeds, resulted in the protection of root diseases from fungal pathogens such as *Fusarium* [23] through different mechanisms such as phosphate solubilization and indole-3-acetic acid (IAA), ammonia, hydrogen cyanide (HCN), and enzyme production [24]. Today, the risk of fungal plant disease is one of the most urgent issues [25,26]. In light of our increased understanding of environmental issues, we must look for feasible and easy-to-use solutions to *Fusarium*’s harmful effects on plants. One of the most famous pathogens of fungal diseases, *F. oxysporum*, hurts crops, especially vegetables [8,27]. Cyanobacteria could be a viable alternative to synthetic fungicides in the fight against phytopathogenic fungi because they produce bioactive metabolites with high antifungal efficiency, particularly polyphenols and flavonoids [28,29]. The use of growth-stimulating cyanobacteria is a common strategy for researchers to enhance and improve the defense capacity and physiological immunity of plants as well as the bioavailability of minerals in the soil [30].

*Anabaena* sp. and *Oscillatoria nigro-viridis* recorded the highest phenolics and flavonoid levels and the most elevated antifungal activity among the investigated cyanobacterial strains in recent research [31]. Accordingly, we examined phenolics and flavonoid levels in the three cyanobacterial strains used in this study to determine which cyanobacterial strains are best for reducing the risk of pepper plant infection with *Fusarium oxysporum*. However, this study’s major purpose was to explore cyanobacteria’s activity to minimize the harmful effect of pepper *Fusarium* wilt by enhancing pepper plant immunity.

## 2. Materials and Methods

### 2.1. Source of Pathogen

The pathogen was received from Al-Azhar University’s Regional Center for Mycology and Biotechnology (RCMB). The pathogen was grown on PDA media and incubated at 28 ± 2 °C for 5 days before being preserved at 4 °C. According to [2], the pathogenic fungus inoculum was ready.

### 2.2. Growth Conditions of Cyanobacteria

This study uses three isolates related to cyanobacteria: *Desmonostoc muscorum* HSSASE1 KT277784, *Anabaena oryzae* HSSASE6 KT277789, and *Arthrospira platensis* HSSASE5 KT277788. Cyanobacterial samples were obtained from the botany and microbiology department, science faculty, Cairo University, Egypt. These strains were previously isolated from Egyptian soil, showing a significant antifungal activity and recording elevated levels of phenolics and flavonoids in vitro. The axenic cultures of the tested isolates were identified and deposited in GenBank under accession numbers according to [32]. Cyanobacterial strains were cultured in BG11 medium [33], but *Arthrospira platensis* was cultivated in Zarrouk medium [34]. A shaker incubator was used to grow all the cyanobacterial strains, which have been maintained in highly controlled growth conditions. Cyanobacterial cultures were incubated under constant illumination of (150 ± 10 μmol photons m^−2^ s^−1^) at 27 ± 2 °C, pH = 7 for *Desmonostoc muscorum*, *Anabaena oryzae*, and at 34 ± 2 °C, pH = 8.7 for *Arthrospira platensis* and a continuous 5% CO_2_ airflow was provided via an air pump. After 14 days, total biomass was harvested at the end of the growth stationary phase by centrifugation at 4200× *g* for 10 min, and then pellets were rinsed with water and lyophilized.

### 2.3. Preparation of Cyanobacteria Extracts

After 14 days of cultivation, freeze-dried cyanobacteria biomass was exposed to aqueous extraction [35]. After washing 100 mg of the cyanobacterial dry biomass in sterile distilled water, the amount was dissolved in 12.5 mL phosphate buffer (0.1 M pH 6.0) for 10 min before sonication (5 s pulses of 8 W over 30 s, on ice). The phosphate buffer solution did not affect extract nutrient composition because it was employed to control and maintain system pH. In addition, the extraction tubes were kept at 4 °C for 24 h. Aqueous extracts were obtained by centrifugation at 8000× *g* for 10 min and then freeze-dried.

### 2.4. Total Phenolic Content (TPC) of Tested Cyanobacteria

Folin–Ciocalteu technique [36] has been employed. For assessment of the total phenolic content of each cyanobacterial strain extract, the Gallic acid (0–500 mg L^−1^) was used to construct a standard calibration curve. The absorbance of the sample was tested to a blank at 760 nm. Gallic acid equivalent (GAE)/g extract was used to measure total phenolic content (TPC).

### 2.5. Total Flavonoids Content (T.F.C.s) of Tested Cyanobacteria

Total flavonoid contents were measured for the tested cyanobacterial strains using a colorimetric method [37]. Five hundred microliters of the cyanobacterial extract were dissolved in 2 mL methanol and then mixed with 3 mL of water, 100 µL of potassium acetate (1 M), and 100 µL of aluminum chloride. After that, the samples were left in the dark for 30 min. The mixture’s absorbance was measured at 415 nm. The total flavonoid content was calculated using a standard curve as (mg/concentration of Quercetin Equivalent (QE) obtained from calibration curve) mg Q.C.E./g of extract.

### 2.6. Experimental Design

The Agricultural Research Center in Giza, Egypt, provided three-week-old pepper seedlings. The consolidated seedlings were transplanted into 40 × 40 cm plastic pots, each treatment containing 6 seedlings. The pots in the green plastic house had a 1:3 mixture of sand and clay, totaling 7 kg. The pots were kept in the greenhouse at a temperature of 22 °C during the daylight hours and 18 °C at the nighttime, with a relative humidity of 70–85%. Except for the healthy control pot, the pathogenic fungus *F. oxysporum* (10^7^ spores mL) was introduced into the soil after planting. For five days, the plants were irrigated routinely. Then, before and after flowering, a one-handed pressure sprayer was used to spray microalgae suspensions to the leaves of the plants three times (20 mL per plant once every week) at a concentration of 1 g L^−1^. Roots were soaked in 1 g of dried algae extract per kg of soil. All plants were irrigated every 72 h for the period of the experiment. The pots were arranged in three duplicates in a completely randomized design: T1-healthy control (sowing pepper seedlings in sterilized soil), T2-infected control (sowing the pepper seedlings in sterilized soil inoculated with *F. oxysporum*), T3-infected plants treated with *Desmonostoc muscorum* through the soil, T4-infected plants treated with *Anabaena oryzae* through the soil, T5-infected plants treated with *Arthrospira platensis* through the soil, T6-infected plants treated with *Desmonostoc muscorum* through the foliar spray, T7-infected plants treated with *Anabaena oryzae* through the foliar spray and T8-infected plants treated with *Arthrospira platensis* through foliar application. For plant resistance evaluation, morphological and biochemical signals from plant samples were analyzed 45 days after sowing, and the disease was assayed.

### 2.7. Disease Symptoms and Disease Index

The disease symptoms were observed 45 days after sowing. The disease index and plant protection were assessed using a score consisting of five classes, as described in [38] with minor adjustments. (1) minor yellowing of lower leaves, (2) moderate yellow plant, (3) wilted plant with browning of vascular bands, and (4) severely stunted and damaged plants. It’s worth noting that the percent disease index (PDI) was determined using a five-grade scale and the formula below. PDI = (1n1+ 2n2 + 3n3 + 4n4)100/4nt, where n1–n4 represents the number of plants in each class and nt represents the total number of plants examined. In addition, the following formula was used to obtain % Protection (P percent): P percent = A − B/A 100 percent, where A is the PDI in infected control plants and B is the PDI in infected plants treated with cyanobacteria.

### 2.8. Resistance Indicators in Pepper Plant

#### 2.8.1. Morphological Resistance Indicators 

Shoot length, root length, and the number of leaves were recorded.

#### 2.8.2. Photosynthetic Pigment Determination

To determine the presence of chlorophyll a, chlorophyll b, and carotenoids in fresh pepper newly leaves (one leaf for each replicate), the photosynthetic pigments were tested according to [39,40]. Throughout this technique, photosynthetic pigments were extracted from fresh leaves (0.5 g) using 50 mL of acetone (80%), then the green color was determined spectrophotometrically at 665, 649, and 470 nm after the extract was filtered.

#### 2.8.3. Estimation of Osmolytes Content

##### Soluble Sugar Determination

The dried shoot’s soluble sugar content was estimated using the method [41,42]. The dried shoots (0.5 g) from each treatment were diluted with 5 mL of 30% trichloroacetic acid (TCA) and 2.5 mL of 2% phenol and filtered through filter paper, then 1 mL of the filtrate was treated with 2 mL of anthrone reagent (2 g anthrone/L of 95% H_2_SO_4_). 620 nm was used to determine the produced blue-green color.

##### Soluble Protein Estimation

The dry shoot’s soluble protein content was estimated using the method described in [43]. 5 mL of 2% phenol and 10 mL of deionized water were used to extract the dried pepper shoots. One mL of this extract was combined with 5 mL of alkaline reagent (50 mL of 2% Na_2_CO_3_ prepared in 0.1N NaOH and 1 mL of 0.5% CuSO_4_ prepared in 1% potassium sodium tartrate) and 0.5 mL of Folin’s reagent (diluted by 1:3 *v*/*v*). After 30 min, a color change could be seen at 750 nm.

##### Proline Content Determination

The proline content of the dried shoot was determined using the technique [44]. The dried shoots (0.5 g) were digested by 10 mL (3%) of sulfosalicylic acid in this technique. In a boiling water bath, 2 mL of the filtrate was mixed with 2 mL of ninhydrin acid and 2 mL of glacial acetic acid for an hour. Then the mixture was placed in an ice bath to stop the reaction. 4 mL of toluene was added to the mix, then the absorbance at 520 nm was determined.

##### Total Phenol

A previous technique [26,45] was used to determine the total phenol content. One g of dried pepper shoots were extracted in 5–10 mL of 80% ethanol for at least 24 h. After dehydrating the alcohol, the remaining residue was extracted thrice using 5–10 mL of 80% ethanol each time. The purified extract was then filled to a capacity of 50 mL with 80% ethanol, and then 0.5 mL of the extract was mixed well with 0.5 mL of Folin’s reagent and shaken for 3 min. After that, 3 mL of purified water and 1 mL of saturated sodium carbonate solution were added and thoroughly mixed. The blue color was detected at 725 nm after 1 h.

#### 2.8.4. Estimation of Malondialdehyde (MDA) and Hydrogen Peroxide (H_2_O_2_) Contents

The method [41] was used to determine the amount of MDA in fresh pepper leaves. New leaf samples (0.5 g) were extracted with 5% TCA and centrifuged for 10 min at 4000× *g*. 2 mL of the extract was combined with 2 mL of 0.6% thiobarbituric acid (TBA) solution and placed in a water bath for 10 min. After cooling, the generated color’s absorbance was measured at 532, 600, and 450 nm. The following equation was used to calculate the MDA content:

6.45 × (A_532_ − A_600_) − 0.56 × A_450_.

Fresh pepper leaves were tested for H_2_O_2_ content [46]. Fresh pepper leaves (0.5 g) were added to 4 mL cold acetone, then 3 mL of the extract was mixed with 1 mL of 0.1% titanium dioxide in 20 percent (*v*:*v*) H_2_SO_4_, then the mixture was centrifuged at 6000× *g* for 15 min. The yellow color generated at 415 nm was detected.

#### 2.8.5. Antioxidant Enzymes Activities Assay

POD and PPO enzyme activity was evaluated in this study to obtain a clear indication of defense-related enzymes. The peroxidase activity (POD) and polyphenol oxidase (PPO) enzymes were assayed according to [38], respectively.

#### 2.8.6. Isozyme Electrophoresis

Peroxidase (POD) isozyme electrophoresis was analyzed according to the technique [26], while polyphenol oxidase (PPO) isozyme was recorded using the method [47]. The Gel Doc VILBER LOURMAT approach was used to evaluate and investigate gels. While the gels were saturated, the banding shape was videotaped, and the band’s number was declared in each gel lane and computed and correlated with each other. The Helena Densitometer Model Junior 24 was used to determine quantitative band quantity and strength changes.

#### 2.8.7. Endogenous Hormones (IAA, SA, and ABA) Contents

Plant hormones are natural organic compounds that affect growth and metabolism, affecting all external manifestations and chemical reactions. These act as chemical signals to activate or inhibit plant growth. As mentioned in [47], the Indole acetic acid (IAA), Salicylic acid (SA), and Abscisic acid (ABA) contents were measured in the terminal buds of both treated and control plants.

### 2.9. Statistical Analysis

Analyses were conducted using one-way variance (ANOVA). The statistically significant differences between treatments with a *p*-value of 0.05 or lower, the LSD test was used with CoStat (CoHort, Monterey, CA, USA) [48].

## 3. Results

### 3.1. Total Phenolics and Flavonoids of the Applied Cyanobacteria

**Figure 1:**
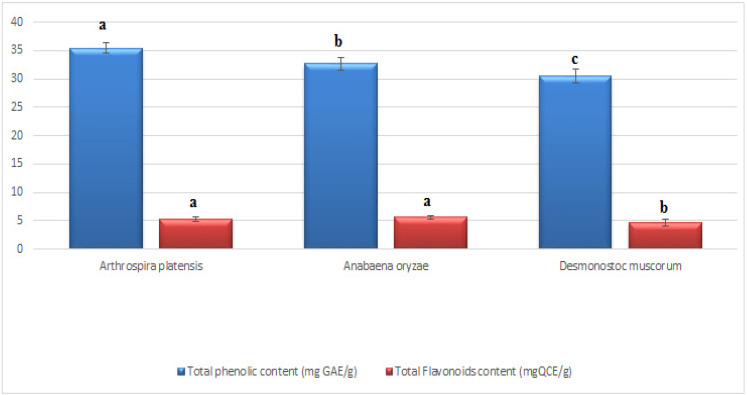
Variation in the total phenolics and flavonoid content of different applied cyanobacteria in the soil application and foliar spraying. Data represent mean ± SD, n = 3. (a–c Letters revered to significant in statically analysis).

The total phenolics and flavonoids of each cyanobacteria species were evaluated at the end of the stationary phase of growth. It can be shown in (Figure 1) that the total phenolics and flavonoids vary with species. The highest total phenolic content was determined in *A. platensis* at 35.39 ± 0.19 mg GAE/g, followed by *A. oryzae* at 32.71 ± 19 mg GAE/g. In terms of flavonoids, *A. oryzae* had the highest content (5.58 ± 0.29 mg QCE/g), followed by *A. platensis* (5.34 ± 0.33 mg QCE/g). On the other hand, *D. muscorum* recorded the lowest phenolics and flavonoid content (30.47 ± 0.31 mg GAE/g and 4.73 ± 0.16 mg QCE/g, respectively).

### 3.2. Effect of Cyanobacteria on Disease Index of Infected Pepper Plants

Data presented in Table 1 reported that *F. oxysporum* shows a highly destructive effect on pepper plants that caused typical wilt symptoms with DI 83.33%. Applying cyanobacterial filtrate in the soil and foliar spraying highly protected plants against *Fusarium* wilt and lowered the *Fusarium* wilt disease symptoms caused by *F. oxysporum* compared with infected pepper plants. Data showed that spraying cyanobacteria was more effective than irrigation in soil, while foliar spraying with *A. platensis* showed high protection against *Fusarium* infection (95%), followed by *D. muscorum* (90%), then *A. oryzae* (69.9%).

### 3.3. Resistance Indicators in Pepper Plant

#### 3.3.1. Morphological Indicators

**Figure 2:**
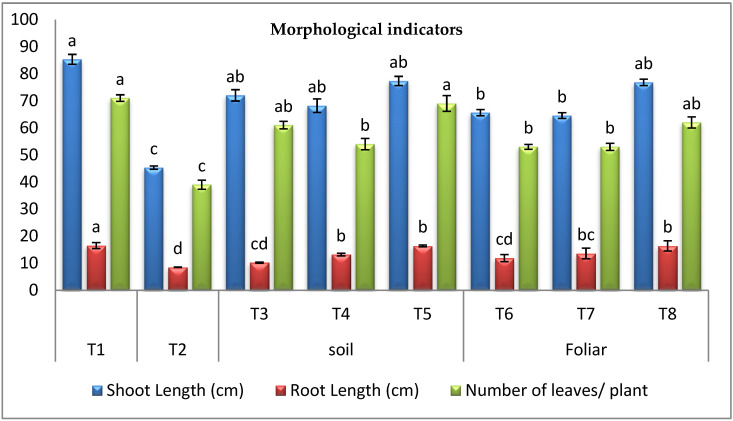
Effect of cyanobacteria on morphological traits of the infected pepper plant with *Fusarium* wilt (T1-healthy control, T2-infected control, T3-infected plants treated with *D. muscorum* soil, T4-infected plants treated with *A. oryzae* soil, T5-infected plants treated with *A. platensis* soil, T6-infected plants treated with *D. muscorum* foliar spray, T7-infected plants treated with *A. oryzae* foliar spray and T8-infected plants treated with *A. platensis* foliar application.) (Data represent mean ± SD, n = 3), (a–d Letters revered to significant in statically analysis).

The results in (Figure 2) indicated that *Fusarium*-wilt hurt all vegetative pepper growth compared to healthy control. *F. oxysporum* minimized shoot length by 46.89%, root length by 48.45%, and the number of leaves by 45.07%, respectively. Regarding the effect of cyanobacteria, it was observed that infected plants treated with *D. muscorum, A. oryzae,* and *A. platensis* through different modes, i.e., soil and foliar spraying showed promising recovery. It was determined that *A. platensis* was the most effective cyanobacteria for recovering plant height by 70.64% and 69.53%, root lengths by 92.94% and 92.94%, and the number of leaves by 76.92% and 58.97% through the soil and foliar application, respectively.

#### 3.3.2. Photosynthetic Pigments

**Figure 3:**
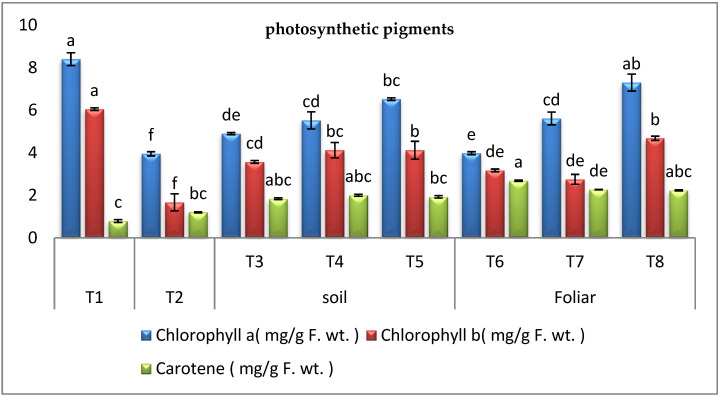
Effect of cyanobacteria on photosynthetic pigments of the infected pepper plant with *Fusarium* wilt (T1-healthy control, T2-infected control, T3-infected plants treated with *D. muscorum* soil, T4-infected plants treated with *A. oryzae* soil, T5-infected plants treated with *A. platensis* soil, T6-infected plants treated with *D. muscorum* foliar spray, T7-infected plants treated with *A. oryzae* foliar spray and T8-infected plants treated with *A. platensis* foliar application.) (Data represent mean ± SD, n = 3), (a–f Letters revered to significant in statically analysis).

Results in (Figure 3) showed that chlorophyll a (Chl a) and b (Chl b) were highly inhibited in *Fusarium* infected pepper plants by 39.41% and 59.46%, respectively. On the other hand, the present study showed that the level of carotenoids in infected pepper plants increased compared to the healthy control. However, the application of cyanobacteria through the soil and foliar application to the infected plants significantly increased the level of carotenoids over the infected control plants. It was found that foliar and soil application of *A. platensis* was the most effective way to enhance infected plants’ levels of Chl a, b (84.81% and 191.01%). On the other hand, *D. muscorum* foliar spraying was the most efficient method for increasing the carotenoids in infected plants (123.00%).

#### 3.3.3. Osmolytes (Soluble Sugar, Soluble Protein, and Proline) Contents

Results in (Table 2) clearly showed *F. oxysporum* infection caused a major reduction in contents of soluble sugars by 38.50% and soluble protein by 57.13% over the healthy control. Still, it caused proline increment by 11.84% over the healthy control. The infected pepper plants treated with cyanobacteria ameliorated total carbohydrates, protein, and proline contents. Cyanobacteria treatment through different modes of application showed a high response. It increased the Accumulation of osmolytes (soluble sugar, soluble protein, and proline) contents compared to the infected control plants.

#### 3.3.4. Stress Biomarkers

**Figure 4:**
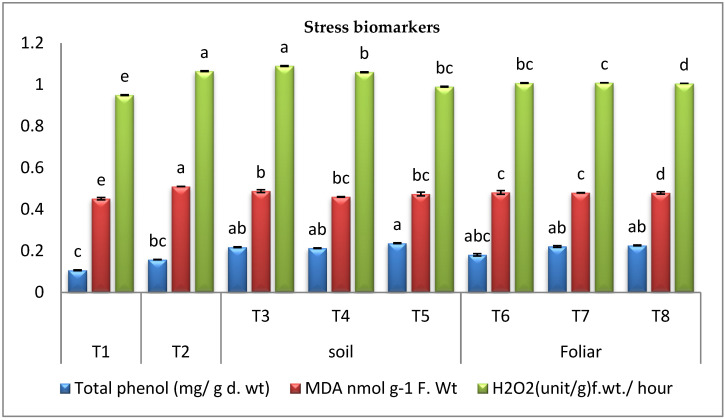
Effect of cyanobacteria on total phenol, Malondialdehyde (MDA), and H_2_O_2_ contents of infected pepper plants (T1-healthy control, T2-infected control, T3-infected plants treated with *D. muscorum* soil, T4-infected plants treated with *A. oryzae* soil, T5-infected plants treated with *A. platensis* soil, T6-infected plants treated with *D. muscorum* foliar spray, T7-infected plants treated with *A. oryzae* foliar spray and T8-infected plants treated with *A. platensis* foliar application.) (Data represent mean ± SD, n = 3), (a–e Letters revered to significant in statically analysis).

The results in (Figure 4) showed that pepper *Fusarium* wilt disease resulted in a rise in phenolics over the healthy control plant. On the other hand, it was observed that infected plants treated with cyanobacteria exhibited a significant increase in phenolics over the infected control plants. The data (Figure 4) indicated that the highest increase in phenolics level was recorded by the infected plants treated with *A. platensis* in the soil treatment. It was observed that cyanobacteria supplementation reduced the generation of H_2_O_2_ and lipid peroxidation (MDA) significantly over the infected control plants (Figure 4). Accumulation of H_2_O_2_ increased in infected control plants, causing an increase in lipid peroxidation (MDA) over the healthy control plants. Supplementation of infected plants with cyanobacteria reduced the generation of H_2_O_2_ over the infected control plants leading to a declined lipid peroxidation (MDA) (Figure 4). The data revealed that the most effective cyanobacterial treatment was foliar spraying with *A. platensis*.

#### 3.3.5. Oxidative Enzymes Activity

**Figure 5:**
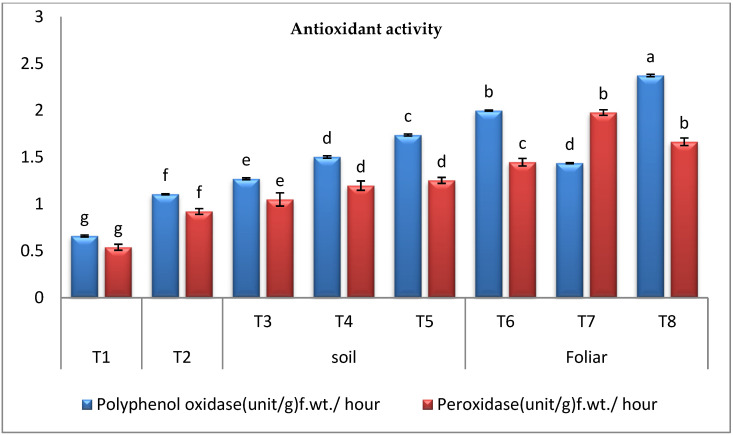
Effect of cyanobacteria on the activity of oxidative enzymes in the infected pepper plants (T1-healthy control, T2-infected control, T3-infected plants treated with *D. muscorum* soil, T4-infected plants treated with *A. oryzae* soil, T5-infected plants treated with *A. platensis* soil, T6-infected plants treated with *D. muscorum* foliar spray, T7-infected plants treated with *A. oryzae* foliar spray and T8-infected plants treated with *A. platensis* foliar application.) (Data represent mean ± SD, n = 3), (a–g Letters revered to significant in statically analysis).

*Fusarium* wilt disease increased the activities of POD and PPO enzymes in the infected control plants over the healthy control plants; however, applying cyanobacteria through different modes to the infected plants significantly enhanced the activities of PPO and POD enzymes over the infected control plants. The data recorded in (Figure 5) revealed that the maximal increase in the activities of PPO and POD enzymes was observed in the infected pepper plants treated with *A. platensis* and *A. oryzae* through the foliar spraying, respectively. According to our findings, the antioxidant enzymes in infected plants treated with cyanobacteria in various modes, such as soil mode and foliar spraying, significantly increased, but the foliar spraying was more effective.

#### 3.3.6. Antioxidant Isozymes

**Figure 6:**
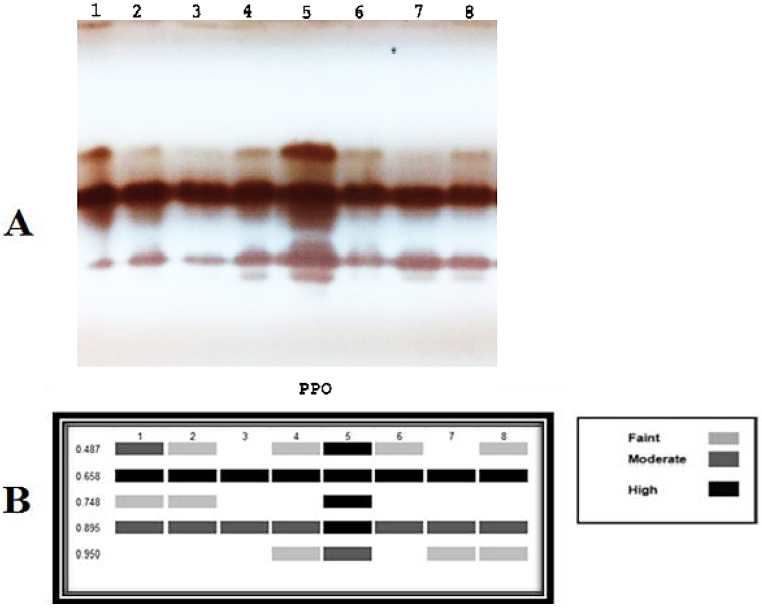
The effect of *F. oxysporum* and the application of cyanobacteria on (**A**) Polyphenol oxidase isozyme. (**B**) Ideogram analysis of PPO isozyme of the infected pepper plants. 1 = Healthy control; 2 = Infected control; 3 = Infected plant + *D. muscorum* (soil); 4 = Infected plant + *A. oryzae* (soil); 5 = Infected plant + *A. platensis* (soil); 6 = Infected plant + *D. muscorum* (foliar); 7 = Infected plant + *A. oryzae* (foliar); 8 = Infected plant + *A. platensis* (foliar).

**Figure 7:**
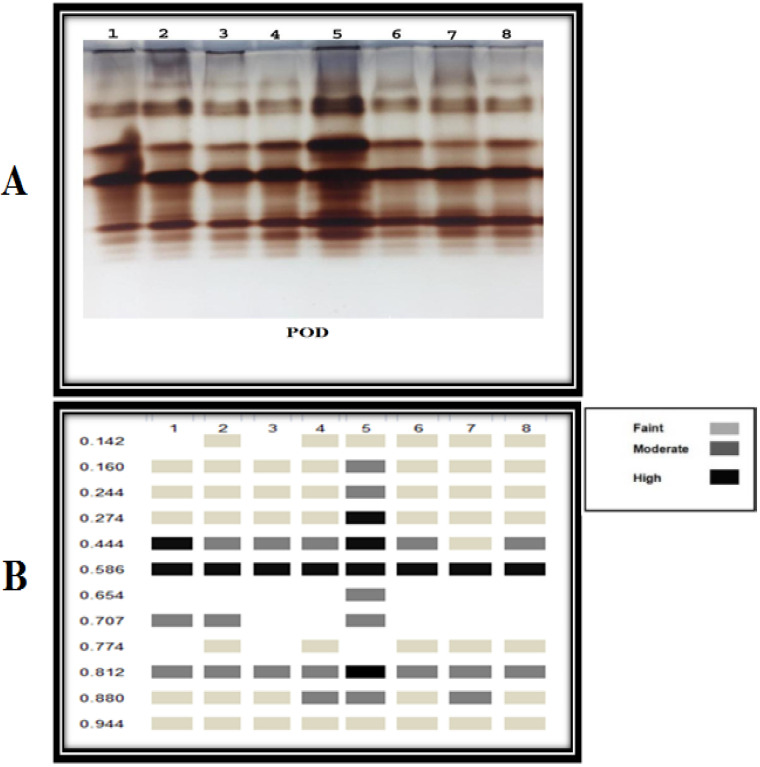
The effect of *F. oxysporum* and the application of cyanobacteria on (**A**) Peroxidase isozyme. (**B**) Ideogram analysis of POD isozyme of the infected pepper plants. 1 = Healthy control; 2 = Infected control; 3 = Infected plant + *D. muscorum* (soil); 4 = Infected plant + *A. oryzae* (soil); 5 = Infected plant + *A. platensis* (soil); 6 = Infected plant + *D. muscorum* (foliar); 7 = Infected plant + *A. oryzae* (foliar); 8 = Infected plant + *A. platensis* (foliar).

The antioxidant isozymes (POD and PPO) could be detected using native PAGE. The results (Figure 6 and Figure 7) revealed the expression of 4 PPO and 9 POD isozymes in the leaves of pepper plants. For PPO isozymes, infected pepper plants treated with *A. platensis* through the soil application showed the strongest PPO expression as it produced 5 bands, including 4 high-intensity bands at Rf (0.487, 0.658, 0.748, and 0.895) and one medium intensity band at Rf (0.950), followed by *A. platensis* through the foliar spraying and *A. oryzae* through the soil application as they showed the same number and density of 4 bands. Referring to POD isozymes, it was observed that treatment of *D. muscorum* (foliar) application showed the strongest POD expression as it produced 11 bands, including four high-intensity bands at Rf (0.274, 0.444, 0.586, and 0.812), five moderated intensity bands at Rf (0.160, 0.244, 0.654, 0.707, and 0.880), and two weak intensity bands at Rf (0.142 and 0.944). In addition, applying cyanobacteria through foliar spraying showed the same number and density of 10 bands for all used cyanobacteria.

#### 3.3.7. Endogenous Hormones

**Figure 8:**
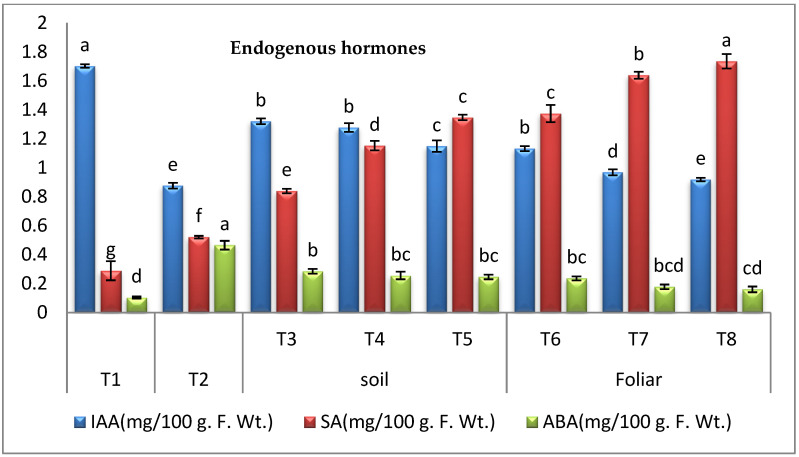
Effect of cyanobacteria on endogenous hormones (IAA, SA, and ABA) of infected pepper plants (T1-healthy control, T2-infected control, T3-infected plants treated with *D. muscorum* soil, T4-infected plants treated with *A. oryzae* soil, T5-infected plants treated with *A. platensis* soil, T6-infected plants treated with *D. muscorum* foliar spray, T7-infected plants treated with *A. oryzae* foliar spray and T8-infected plants treated with *A. platensis* foliar application.) (Data represent mean ± SD, n = 3), (a–g Letters revered to significant in statically analysis).

The results in (Figure 8) highlighted that *Fusarium* infection decreased IAA but increased SA and ABA contents in the infected control plants over the healthy control plants. However, applying cyanobacteria through different modes to the infected plants significantly increased the levels of IAA and SA hormones but decreased ABA concentrations over the infected control plants. The data (Figure 8) revealed that the maximal increase in the IAA contents was observed in the infected plants treated with *D.* *muscorum* and *A. oryzae* through the soil treatment mode. In addition, the highest growth in the SA contents was recorded by the infected plants treated with *A. platensis* through the foliar spraying. On the other hand, infected plants treated with *A. platensis* through foliar spraying showed the least reduction in ABA concentration in our research.

## 4. Discussion

Disease severity was the first guide to govern systemic resistance in treated plants by cyanobacteria. Data presented in this study reported that *F. oxysporum* shows a highly destructive effect on pepper plants that caused typical wilt symptoms with DI 83.33%, similar to earlier studies on the same pathogenic fungus [40]. Using cyanobacterial extract to treat *Fusarium* wilt-infected pepper plants greatly reduced the symptoms of the disease, which is the primary criterion for assessing resistance in the pepper plant. Data showed that foliar spraying with cyanobacteria extracts was more effective than putting them in soil; foliar spraying with *A. platensis* showed high protection against *Fusarium* infection (95%), followed by *D. muscorum* (90%), then *A. oryzae* (69.9%), these results are consistent with [49], which stated that foliar spraying was more effective than soil application, where the nutrients in the foliar spray are absorbed up directly by the leaves of the plant.

Moreover, the results in this study revealed that the highest total phenolic and flavonoid contents were determined in *Arthrospira platensis,* which provided the pepper plant with the highest percentage of protection through foliar spraying. This result may recommend choosing cyanobacterial strains with elevated levels of phenolics and flavonoids to stimulate immune responses in pepper plants against *Fusarium* wilt. The results explained that spraying with cyanobacteria caused an improvement in pepper plant resistance against biotic stresses, including fungal pathogens [49]. Furthermore, antifungal activity of *A. platensis* [50], *D. muscorum* [51], and *A. oryzae* [18] against fungal pathogens were also reported. Reducing symptoms and severity of infection is one of the most important goals of using fungicides, whether chemical or biological [52]. Undoubtedly, applying natural or biological factors such as cyanobacteria is more environmentally friendly. Interestingly, the use of cyanobacteria understudy led to a reduction in symptoms, and a reduction in the severity of infection was reflected positively on the health of plant growth. These results are consistent with several scientific studies [25,53] that confirm that cyanobacteria contain many antioxidants, proteins, vitamins, hormones, and antimicrobials.

The results indicated that *F. oxysporum* hurt all vegetative pepper growth compared to healthy control. *F. oxysporum* minimized shoot length by 46.89%, root length by 48.45%, and several leaves by 45.07%, respectively. Therefore, the infection of the pepper plant with *F. oxysporum* caused a significant inhibition of all growth parameters, where our findings align with those of a huge number of other researchers [3,54]. Regarding the effect of cyanobacteria, it was observed that infected plants treated with *D. muscorum*, *A. oryzae,* and *A. platensis* in soil and foliar spraying exhibited promising recovery. It was determined that *A. platensis* was the most effective cyanobacteria for recovering plant height by 70.64% and 69.53%, root lengths by 92.94%, 92.94%, and the number of leaves by 76.92%, and 58.97% through the soil and foliar application, respectively. These results agree with a previous study [21], which reported that the application of *A. platensis* improved plant growth through polysaccharides production. The use of cyanobacteria to enhance crop growth has been proposed as a potential executive performance in crop enhancement. [55]. These results align with [35], who found that treating plants with cyanobacteria greatly improved their vegetative growth. The increase in vegetative growth and crop yield with cyanobacteria could mainly be due to the release of plant nutrients like N, P, and K and the excretion of plant growth regulators (auxin, gibberellins), amino acids, and vitamins [56].

After applying cyanobacteria, photosynthetic pigments had become a clear positive indicator of sufficient treatments. By analyzing data from this investigation, it was obvious that chlorophylls a and b (Chl a and b) were severely inhibited in *Fusarium* infected pepper plants by 58.22% and 59.46%, respectively. This reduction in chlorophyll was induced by the production of reactive oxygen species (ROS) after the attack with *Fusarium*, which destroyed chlorophyll contents, preventing the plants from capturing sunlight and reducing photosynthesis [38,57]. Chlorophyll disruption, reduced chlorophyll synthesis, and thylakoid membrane strength are also diminished [58]. On the other hand, the present study showed that the level of carotenoids in pepper plants increased by 70% in response to *Fusarium* infection. These findings are consistent with those of other studies [3,6,39,59,60], which found that the content of carotenoids in plants increased dramatically in response to *Fusarium* infection. In terms of cyanobacteria’s beneficial impacts, it was discovered that diseased plants improved after applying *D.* *muscorum*, *A. oryzae*, and *A. platensis* through various modalities. It was found that foliar and soil application of *A. platensis* was the most effective way to enhance infected plants’ Chl a, b, and carotenoids levels. Increased chlorophyll contents in infected plants treated with cyanobacterial strains could be resulted from the higher amount of atmospheric nitrogen assimilation by cyanobacteria then transported to pepper plant tissues [59].

Moreover, cyanobacteria supply decreased ethylene production and chlorophyll, stimulated the synthesis of carotenoids which defend chlorophyll from oxidation, and increased chlorophyll content [60]. These results are in agreement with the results reported in this study. Photosynthetic protection may have been supplied by improved synthesis of carotenoids due to enhancing ROS scavenging [61].

In the current study, the results clearly showed *F. oxysporum* infection caused a major reduction in contents of soluble sugars by 38.50% and soluble protein by 57.13% over the healthy control. Still, it caused proline increment by 11.84% over the healthy control. These results agree with the previous studies [3,62]. The infected pepper plants treated with cyanobacteria showed amelioration in the contents of total carbohydrates, protein, and proline. These results may be explained by the potency of cyanobacteria to secrete complex heteropolymers, polysaccharides, and lipopolysaccharides to induce defense-related gene expression [63,64]. Cyanobacteria-treated plants were capable of fighting against *Fusarium* infection by accumulating more proline, which protects proteins from oxidation [65]. The osmolytes (soluble sugar, proline, and soluble protein) levels in the cyanobacteria-treated plants were significantly higher than those in the infected control plants. Also, oil organic carbon, nutrient absorption, and nitrogen fixation were all improved due to the cyanobacteria treatment [66]. As a result, our findings reveal that osmolyte content significantly increased when infected plants were treated with cyanobacteria, as previously explained [49,62]. This increase in soluble proteins can be explained by activating plant defense systems when pathogens are challenged.

According to the findings of this research, pepper *Fusarium* wilt disease resulted in a rise in phenolics over the healthy control plant. On the other hand, it was observed that infected plants treated with cyanobacteria exhibited a marked increase in phenolics over the infected control plants. The present data indicated that the highest growth in phenolic level was recorded by the infected plants treated with *A. platensis* in the soil treatment. The increased accumulation of phenolics by treatment with cyanobacteria resulted in stress tolerance of the pepper plant against *Fusarium* infection [66,67]. It was observed that cyanobacteria supplementation reduced the generation of H_2_O_2_ and lipid peroxidation (MDA) significantly over the infected control plants. Accumulation of H_2_O_2_ increased in infected control plants, causing an increase in lipid peroxidation (MDA) over the healthy control plants. Supplementation of infected plants with cyanobacteria reduced the generation of H_2_O_2_ over the infected control plants leading to a declined lipid peroxidation (MDA). The data revealed that the most effective cyanobacterial treatment was foliar spraying with *A. platensis*. The findings here are comparable to a previous study conducted by [26], which reported that the application of biological stimulators under stress conditions decreased MDA.

The activity of POD and PPO was assessed to identify enzymes involved in protecting the infected plant. *Fusarium* wilt disease increased the activities of POD and PPO enzymes in the infected control plants over the healthy control plants; however, the application of cyanobacteria through different modes to the infected plants significantly enhanced the PPO and POD enzyme activity over the infected control plants. The recorded data revealed that the maximal increase in the activities of PPO and POD enzymes was detected in the infected pepper plants treated with *A. platensis* and *A. oryzae* through the foliar spraying, respectively. Protective enzymes such as POD and PPO are the most significant in biotic stress response [68]. These enzymes are involved in the early stages of plant resistance to various stressors and the synthesis of phenolic compounds. According to our findings, the antioxidant enzymes in infected plants treated with cyanobacteria in multiple modes, such as soil mode and foliar spraying, significantly increased. The plant displayed distinct strategies for coping with stress by increasing the activity level of some antioxidant enzymes in the cell to maintain a low concentration of reactive oxygen species [26,69].

By detecting the antioxidant isozymes (POD and PPO) by native PAGE, the results showed that a significant part of the plant’s response to various stresses is the isozyme substance, which also serves as an important metabolic regulator. Isozyme is a clear indication of the occurrence of resistance, as it plays an important role in mitigating or limiting free radicals that result from oxidative explosions. The findings of this research fully agree with the other scientific reports [70,71]. In the presence of a *Fusarium* wilt disease, cyanobacteria can induce gene expression in infected plants similarly to antioxidant enzymes. It can produce chemicals activating plant immunity, such as phenols and natural hormones [63].

In this study, results highlighted that *Fusarium* infection decreased IAA but increased SA and ABA contents in the infected control plants over the healthy control plants. However, applying cyanobacteria through different modes to the infected plants significantly increased the levels of IAA and SA hormones but decreased ABA concentrations over the infected control plants. Plant hormones are natural organic compounds that affect growth and metabolism, thereby affecting all external manifestations and chemical reactions. These act as chemical signals to activate or inhibit plant growth. When plants are exposed to infections, a considerable modulation in the biosynthesis of hormones occurs, affecting various growth processes [38]. Phytohormones produced by the cyanobacteria have an important function in controlling plant fungal diseases, activating several genes responsible for systemic resistance in plants [49]. The modulation of plant hormone contents is essential in plant defense reactions against *Fusarium* wilt [72]. The SA plays an important role as a plant hormone, as it stimulates growth and works to activate the chemical and synthetic resistance of the plants against any pathogen, increases the absorption of nutrients, and increases the process of photosynthesis [73,74]. Recently, SA has been used externally or internally as inducers against various plant pathogens [75,76]. SA has been described as a key molecule in the signal transduction pathways of the biological stress response by using cyanobacteria to activate and increase SA, IAA as these hormones act as antimicrobial substances [76,77]. The significance of ABA in plant disease resistance is unclear when compared to that of the plant hormones jasmonic acid and salicylic acid, both of which play key roles in disease resistance [78]. Similarly to our findings, previous research has recorded the accumulation of ABA during the infection of sugar beets by fungi [79].

## 5. Conclusions

It is concluded that *Fusarium* wilt generated oxidative destruction and developed into reduced growth and dropped physiological performance. Applying cyanobacteria to *Fusarium* infected pepper plants through soil application or foliar spraying activated the immunity of infected pepper plants. The infected plants treated with cyanobacteria showed enhanced photosynthetic pigments and accumulation of osmoprotectants, phenols, and antioxidant systems that also act as a scavenging tool to remove the excess ROS under *Fusarium* infection. Therefore, it could be applied in agricultural fields through soil or foliar application. As far as we know, this is the first evidence to report that cyanobacteria metabolites influence the isozymes of pepper plants attacked with *Fusarium*, and supplementary molecular findings can provide information on the impact of cyanobacteria on the metabolism of the plant under biotic stress. This study supports the positive application of cyanobacteria in protecting pepper plants under fungal infection; however, further studies are required to unravel actual mechanisms.

## Figures and Tables

**Table 1:** Effect of cyanobacteria on disease index of the infected pepper plant with *Fusarium* wilt.

Treatments	Method of Application	Disease Symptoms Classes					DI (Disease Index) (%)	Protection (%)
		0	1	2	3	4		
*D. muscorum*	Through soil	2	1	2	0	1	37.5	54.99
*A. oryzae*		1	3	1	1	0	33.33	60.03
*A. platensis*		2	1	3	0	0	29.16	65
*D. muscorum*	Through Foliar	5	0	1	0	0	8.3	90
*A. oryzae*		3	0	3	0	0	25	69.9
*A. platensis*		5	1	0	0	0	4.16	95
Control infected		0	0	1	2	3	83.33	0

**Table 2:** Effect of cyanobacteria on osmolytes (soluble sugar, soluble protein, and proline) mg g^−1^ DW) contents of the infected pepper plant with *Fusarium* wilt. Data presented as means ± SD (n = 3). Data followed by letters are significantly different in the LSD test at *p* ≤ 0.05.

Treatments	Method of Application	Total Carbohydrate	Total Protein	Free Proline
Healthy control		30 ± 2.4 ^a^	34.2 ± 0.2 ^a^	0.76 ± 0.002 ^d^
Infected control		18.45 ± 1.62 ^c^	14.66 ± 1.6 ^f^	0.85 ± 0.001 ^e^
*D. muscorum*	Through soil	19.55 ± 0.82 ^c^	29.08 ± 0.4 ^b^	0.913 ± 0.001 ^a^
*A. oryzae*		27.7 ± 0.9 ^a^	22.26 ± 1 ^e^	0.86± 0.001 ^b^
*A. platensis*		24.02 ± 2.62 ^b^	29.56 ±0.3 ^b^	0.91 ± 0.002 ^a^
*D. muscorum*	Through Foliar	19.74 ± 1.4 ^c^	26.46 ± 0.9 ^c^	0.862± 0.001 ^c^
*A. oryzae*		20.7 ± 1.7 ^c^	24.8 ± 0.7 ^d^	0.863 ± 0.001 ^c^
*A. platensis*		21.38 ± 1.26 ^bc^	28.56 ± 0.7 ^b^	0.864 ± 0.001 ^bc^
LSD at 0.05		2.967	1.507	0.0029

## Data Availability

The data presented in this study are available in the article.
